# Dyslipidemia and its associated factors among adult cardiac patients at Ambo university referral hospital, Oromia region, west Ethiopia

**DOI:** 10.1186/s12872-023-03348-y

**Published:** 2023-06-24

**Authors:** Bedasa Addisu, Shiferaw Bekele, Temesgen Bizuayehu Wube, Agete Tadewos Hirigo, Waqtola Cheneke

**Affiliations:** 1 Department of Medical Laboratory Science, School of Medicine, College of Medicine and Health Sciences, Debre Berhan University, PO Box 445, Debre Berhan, Amhara Ethiopia; 2 School of Medical Laboratory Sciences, Faculty of Health Sciences, Institute of Health, Jimma University, Jimma, Ethiopia; 3 School of Medical Laboratory Science, College of Medicine and Health Sciences, Hawasa University, Hawasa, Ethiopia

**Keywords:** Dyslipidemia, Cardiovascular diseases, Ambo, Ethiopia

## Abstract

**Background:**

Cardiovascular disease is a cluster of illnesses that affect the heart and blood vessels. Dyslipidemia is the most common risk factor for cardiovascular disease, causing more than 4 million deaths each year worldwide. However, there is very little evidence concerning the prevalence and pattern of dyslipidemia among cardiac patients in Ethiopia.

**Methods:**

Hospital-based cross-sectional study was conducted from June to September 2022 at Ambo University referral hospital. Data on socio-demographic, clinical and anthropometric features were collected from adults with cardiac diseases using a convenient sampling technique. Lipid profiles and uric acid were measured from overnight fasting blood. The national cholesterol education program adult treatment panel (NCEP-ATP) III criteria was used to define dyslipidemia.

**Results:**

A total of 269 participants were enrolled and the overall 76.6% [95% confidence interval (CI):72.1–81] of patients had at least one dyslipidemia. The prevalence of total cholesterol (TC) ⩾200 mg/dl, triglyceride (TG), LDL-cholesterol and HDL-cholesterol < 40 mg/dl were 38.9%, 44.6%, 29.4%, and 53.5%, respectively. Age > 54 was associated with TC and TG dyslipidemia, adjusted odds ratio (aOR) and (95% CI) were 2.6(1.4–4.8) and 2.4(1.2–4.7), respectively. While, a family history of heart disease, sedentary lifestyle and obesity were associated with TC dyslipidemia, aOR (95%CI) were 1.9(1.1–3.5), 1.4 (1.4–14.6) and 6.7 (1.4–32.5), respectively. In addition, diabetetes mellitus and abdominal obesity were significantly associated with TG dyslipidemia, aOR (95%CI) were 1.9(1.0–3.6) and 2.6(1.16–5.8), respectively. Moreover, uric acid was positively correlated with TC and TG level.

**Conclusions:**

The results indicate that more than 75% of the cardiac patients had at least one dyslipidemia. This reflects the need for regular monitoring of lipid profiles and intensive counseling in this population to mitigate further cardio-metabolic complications.

## Introduction

Cardiovascular diseases(CVDs) are a group of disorders that damage the cardiovascular system such as heart, blood vessels, and circulatory system [1, 2]. Due to increased urbanization and lifestyle changes that increase exposure to risk factors caused by changes in nutrition, physical activity, and environment, the disease remains the leading cause of death worldwide [3]. CVD caused 17.9 million deaths worldwide in 2019, accounting for 32% of all global deaths [4]. The most important risk factors for CVD are dyslipidemia, obesity, hypertension, diabetes mellitus (DM), unhealthy diet, physical inactivity, smoking, alcohol consumption, aging, and a family history of cardiac diseases [4–6].

Dyslipidemia is the most important modifiable risk factor of the cardiovascular disease. It can be defined as an increasing concentration of lipids and lipoproteins in the blood, either individually or in combination [7, 8]. Lipids play a key role in the development and progression of atherosclerosis, leading to clinical consequences such as stroke, heart failure, myocardial infarction and kidney failure. Atherosclerosis begins with endothelial cell dysfunction/damage [9]. Atherogenic dyslipidemia such as elevated LDL-cholesterol (LDL-c), low HDL-cholesterol (HDL-c) and high triglyceride (TG) concentrations have been implicated as the possible risk factors for CVD [10]. In addition, cholesterol is deposited into the arterial intima and leads to endothelial cell lesions which induce the release of reactive oxygen species that trigger oxidative stress which leads to impaired left ventricular function. LDL-c oxidation and structural modification increase the permeability of endothelial cells which involves the expression of adhesion molecules, chemotactic proteins, and growth factors for monocyte-macrophages. After subsequently invading beneath the endothelium, monocytes changed into macrophages, accumulate oxidized lipoproteins and turn into foam cells. Lipid accumulation also causes smooth muscle cell proliferation and inflammatory cell activation that eventually, leads to necrosis and plaque development, which result in atherosclerosis [9, 11, 12].

Hypertriglyceridemia combined with high LDL-c significantly increases the risk of coronary artery disease (CAD). World health organization (WHO) estimates of 2012 showed that dyslipidemia accounted for 18% of ischemic heart disease, 56% of stroke, and more than 4 million deaths per year globally [8]. Patients with hyperlipidemia are about twice as likely to develop CVD [13]. A population-based study conducted by Nieto et al. indicated that only 42% of the population had been aware and informed about their hypercholesterolemia and only 4% were receiving lipid-lowering agents [14]. In 2017, the report indicated that about three hundred thousand people were affected by CVD in Ethiopia [15]. Hypercholesterolemia and hypertriglyceridemia were found almost in one-third of the CVD patients [16], 34% of patients with hypertensive heart disease had a lipid abnormality [17] and 63% of patients with CAD had dyslipidemia in Ethiopia [18].

The burden of dyslipidemia can vary from person to person and geographical location depending on age, disease, and environment, dietary and lifestyle-related factors. The frequent bunching of dyslipidemia and other CVD risk factors in cardiac patients has been shown to act synergistically, accelerating the development of atherosclerosis and cardiovascular morbidity and mortality. Identifying the potential contributory factors of dyslipidemia in CVD patients is crucial in order to manage the disease condition and reduce further complications. Therefore, this study aimed to fill this gap by assessing the prevalence, pattern, and associated factors of dyslipidemia among patients with CVDs.

## Methods

### Study setting and study population

The study was conducted at Ambo university referral hospital (AURH) in west Ethiopia. AURH was established in 2016 and situated in Ambo town which is the capital of the west Shewa zone, 114 km away from Addis Ababa (the capital city of Ethiopia). A hospital-based cross-sectional study was conducted among adult patients with cardiac disease. Adults who had cardiac problems with age ≥ 18 years old were eligible for the study. However, patients using lipid-altering drugs, women with confirmed pregnancy, previously diagnosed HIV patients and receiving antiretroviral agents, patients with serious illness, and patients with known chronic liver and renal failures were all excluded from the study.

### Sample size and sampling technique

The sample size was estimated using single population proportion formula considering 63% of dyslipidemia in CAD patients [18] with a 95% confidence interval (CI) and a 5% margin of error.

$$\mathrm{n}=\frac{{\left(\mathrm{Z}\raisebox{1ex}{$\mathrm{\alpha }$}\!\left/ \!\raisebox{-1ex}{$2$}\right.\right)}^{2}\times \mathrm{pq}}{{\mathrm{d}}^{2}}$$

where, *P* = proportion of dyslipidemia, Zα/_2_ = critical value at 95%, level of confidence (Z = 1.96), d = margin of error (5%), *n* = the required sample size, which is 358. However, the number of source populations is < 10,000; sample size correction was performed using Cochran's formula for sample size correction. A final sample size of 269 was then calculated. Finally, a consecutive convenient sampling technique was applied to select and include the study participants.

### Assessments and measurements

Socio-demographic, clinical and other relevant information was collected through pre-tested structured questionnaires administered by face-to-face interviewers. While, anthropometric measurements such as weight, height, body mass index (BMI) and waist circumference were taken by trained professional nurses working at the chronic diseases clinic, based on the WHO step-by-step approach to surveillance of non-communicable disease risk factors [19]. The height and weight of each subject were measured using ASTOR adult scale, with subjects wearing light clothes and no shoes. The BMI was calculated by dividing weight (in kilograms) by height square (in meter^2^) and the results were recorded. Then BMI was categorized as underweight (BMI < 18.5 kg/m^2^); normal weight (BMI = 18.5 to 24.9 kg/m^2^); overweight (BMI = 25 to 29.9 kg/m^2^); and obese (BMI ≥ 30 kg/m^2^ [20].

While the waist circumference (WC), on the other hand, was measured at the midpoint between the iliac crest and the lower rib with non-elastic measuring tape. Blood pressure was measured with a mercury sphygmomanometer (UM-211 blood pressure monitor) and the accuracy of the measurement was maintained by measuring at least two readings within 3–5 min of difference after patients resting in the clinic for a minimum of 10–15 min and finally the mean blood pressure (BP) was recorded. Moreover, the global physical activity questionnaire of the STEPS instrument was used to assess physical activity status [19].

A 4–5 ml of venous blood sample was collected from each subject after overnight fasting and COBAS-311**(**Roche, Germany) automated clinical chemistry analyzer was used to determine lipid profile and uric acid from serum sample. The enzymatic colorimetric assay method was used to determine total cholesterol using cholesterol oxidase, phenol 4-amino antipyrine, and peroxidase (CHOD-PAP) reagents; and triglyceride using glycerol phosphatase, phenol 4-antipyrine and peroxidase(GPO-PAP) regents. In addition, LDL-c was measured using cholesterol reaction along with reagents that block the contribution of HDL-c and very low density lipoprotein (VLDL), while the HDL-c was measured by a homogenous enzymatic colorimetric method that used polyanions and a detergent that blocks non-HDL-c lipoproteins. Uric acid was measured by enzymatic colorimetric test in which the uricase enzyme cleaves uric acid to form allantoin and hydrogen peroxide. In the presence of peroxidase, 4-aminoantipyrine is oxidized by hydrogen peroxide to a quinone-diimine dye. Then the color intensity of the quinoneimine formed is directly proportional to the uric acid concentration.

### Definition of dyslipidemia and others

*Dyslipidemia*: was defined according to national cholesterol education program adult panel III (NCEP-ATP III) guidelines, cut-off points that place an individual at risk for cardiovascular disease is: TC ≥ 200 mg/dl, HDL-c < 40 mg/dl, LDL-c ≥ 130 mg/dl and TG ≥ 150 mg/dl [21], whereas hyperuricemia was assessed based on uric acid levels ≥7 mg/dl in males and ≥ 6.0 mg/dl in females [22].

*Abdominal obesity:* men with a WC ≥ 102 cm or women with a WC ≥ 88 cm [20].

*Low fruit/ vegetable intake*: was defined as Intake of seven-day history was used and < 4-day use of fruit and vegetables in a week [23].

### Statistical analysis

The data from each questionnaire were checked visually and entered into Epi-Data version 4.6 (Epi-Data, Odense, Denmark). And then exported and analyzed using the Statistical Package for Social Science (SPSS), version 25. Descriptive statistics such as frequency and percentages were applied to summarize categorical variables. While means and standard deviation also was used for continous data. The chi-square test was used to evaluate the significance of categorical variables with the study outcome; while Pearson’s correlation coefficient was used to find out correlations between the lipid profiles and different independent variables. Both bivariate and multivariable binary logistic regression models were used to evaluate the association of independent factors with outcome variables. Moreover, only a variable with a *p*-value < 0.25 in bivariate analysis was considered for multivariable analysis and finally a *p*-value < 5% was accepted as statistical significance.

### Data quality management

The quality of data was maintained by pre-testing of 10% questionnaires at Meti Health Center prior to actual data collection and then all required amendment was done on questionnaires following a pre-test feedback. Appropriate guidance was given to the data collectors by the principal investigator on how to collect all relevant data for the study purpose. In addition, all laboratory procedures were performed strictly in accordance with the standard operating procedures. The proper functioning of instruments, laboratory reagents and technical performance were checked daily through performing quality control.

## Results

### Socio-demographic and other characteristics of the study subjects

A total of 269 adults with known cardiac disease were included in the study. Of all the study participants, 153(56.9%) were males. The mean age (± SD) of the study subjects was 51.13 ± (15.83) ranging from 18 to 89 years. One hundred sixty-three (60.6%) of the study participants were urban dwellers. The majority (141 = 52.4%) of the study participants had an educational status above secondary school, while 59(21.9%) were unable to read and write. Concerning occupation, ethnicity and marital status; the majority 76(28.3%), 189(70.3%), and 164(61%) were farmers, Oromo and married, respectively.

Forty-six (17%) and 59(21.9%) of the study participants had a history of smoking and alcohol consumption, respectively. In addition, 18 (6.7%) and 28(10.4%) were currently smoking and drinking alcohol, respectively. One hundred ninety (70.6%) of the study participants had less active to sedentary lifestyles. Moreover, one hundred sixty-two (60.2%) of the study participants had low fruit/vegetable consumption habit (Table 1).

**Table 1:** Socio-demographic and other characteristics of the study population

Variables	Category	Dyslipidemia		
		Frequency (%)	Yes, n (%)	No, n (%)
Age	18–25	28(10.4)	22(8.2)	6(2.2)
	25–34	13(4.8)	6(2.2)	7(2.6)
	35–44	43(16.0)	30(11.1)	13(4.9)
	45–54	64(23.8)	46(17.1)	18(6.7)
	> 54	121(45.0)	102(37.9)	19(7.1)
	Total	269(100.0)	206(76.6)	63(23.4)
Sex	Male	153(56.9)	118(43.8)	35(13.1)
	Female	116(43.1)	88(32.7)	28(10.4)
Residence	Rural	106(39.4)	77(28.6)	29(10.8)
	Urban	163(60.6)	129(50)	34(12.6)
Education Status	Illiterate	59(21.9)	47(17.5)	12(4.4)
	Primary school	69(25.7)	48(17.8)	21(7.9)
	Secondary school	91(33.8)	71(26.4)	20(7.4)
	Higher education	50(18.6)	40(14.8)	10(3.8)
Occupation status	Student	19(7.1)	18(6.7)	1(0.4)
	Merchant	46(17.1)	34(12.6)	12(4.5)
	Farmer	76(28.3)	52(19.3)	24(9)
	Government employee	66(24.5)	52(19.3)	14(5.2)
	Non-employed	42(15.6)	34(12.6)	8(3)
	Retired	20(7.4)	16(5.9)	4(1.5)
Marital status	Single	39(14.5)	29(10.8)	10(3.7)
	Married	164(61.0)	120(44.6)	44(16.4)
	Separated/divorced	28(10.4)	26(9.7)	2(0.7)
	Widowed	38(14.1)	31(11.5)	7(2.6)
History of smoking	Yes	46(17.1)	35(13)	11(4)
	No	223(82.9)	171(63.6)	52(19.3)
Currently smoking	Yes	18(6.7)	15(5.6)	3(1.1)
	No	251(93.3)	191(71)	60(22.3)
Passive smoker	Yes	64(23.8)	49(18.2)	15(5.6)
	No	205(76.2)	157(58.4)	48(17.8)
History of alcoholism	Yes	59(21.9)	52(19.3)	7(2.6)
	No	210(78.1)	154(57.2)	56(20.8)
Current alcoholism	Yes	28(10.4)	25(9.3)	3(1)
	No	241(89.6)	181(67.3)	60(22.3)
Physical exercise	Vigorous intensity	26(9.6)	14(5.2)	12(4.4)
	Moderate intensity	53(19.7)	32(12)	21(7.7)
	Less active	102(37.9)	76(28.3)	26(9.7)
	Sedentary	88(32.7)	84(31.2	4(1.5)
Fruit/vegetable consumption	Low consumption	162(60.2)	122(45.3)	40(14.9)
	Sufficient consumption	107(39.8)	84(31.2)	23(8.6)

### Anthropometric and clinical characteristics of the study participant

The mean (± SD) of SBP, DBP, BMI, and WC of the study participants were 132.83 ± 14.9, 85.5 ± 7.4, 24.39 ± 4.02, and 91.42 ± 10.4, respectively. More than half (140 = 52%) of the study participants had normal weight, 68(25.3%) were overweight, 43(16%) were obese and the remaining 18(6.7%) were underweight. In addition, 148(55%), 77(28.6), and 106(39%) of study participants had a history of hypertension, DM and a family history of cardiovascular disease (FHHD), respectively. The mean (± SD) of participants’ time since heart disease and treatment experience were 3.88 ± 2.134 and 3.23 ± 1.8 years, respectively.

### The pattern dyslipidemia in relation to different variables and cardiac disease type

Overall 206(76.6%; 95% CI (72.1–81.0)) of the study participants had at least one lipid profile abnormality that is compatible with a diagnosis of dyslipidemia according to NCEP-ATP III guidelines. The prevalence of TC ≥ 200 mg/dl, TGs ≥ 150 mg/dl LDL-c ≥ 130 mg/dl and HDL-c < 40 mg were 39.8%, 44.6%, 29.4%, and 53.5%, respectively. TC dyslipidemia was higher in males than females, but the difference was not statistically significant (41.1% vs. 37.9%, *p* = 0.59), while LDL-c dyslipidemia was marginally higher in females than males (34.5% vs. 25.5% *p* = 0.11), respectively. In addition, participants aged > 54 years had significantly higher TC (48.7% vs. 32.4, *p* = 0.006) and TG (56.2% vs. 35%, *p* = 0.001) than participants aged ≤ 54 years. The prevalence of TC, TG, and LDL-c-dyslipidemia was significantly higher in participants with abdominal obesity than participants with normal WC. Moreover, TC, TG, and LDL-c dyslipidemia were higher in participants with a sedentary lifestyle, while HDL-c dyslipidemia was higher in participants who perform less physical activity. TC and HDL-c dyslipidemia were significantly higher in participants with a history of DM than non-DM (58.4% vs. 39%, *p* = 0.004) and (64.9% vs. 48.9%, *p* = 0.018) (Table 2).

**Table 2:** Pattern of dyslipidemia in relation to different variables among cardiac patients

Variable	Category	Frequency	Outcome variables			
			TC ≥ 200 mg/dl	TGs ≥ 150 mg/dl	LDL-c ≥ 130 mg/dl	HDL-c < 40 mg/dl
Sex	Male	153(56.8)	63(41.2)	69(45.1)	39(25.5)	84(54.9)
	female	116(43.2	44(37.9)	51(43.9)	40(34.5)	60(51.7)
	***P-value***		*0.59*	*0.85*	*0.11*	*0.60*
Age	≤ 54	148(55)	48(32.4)	52(35)	39(26.3)	76(51.3)
	> 54	121(45)	59(48.7)	68(56.2)	40(33)	68(56.2)
	***P-value***		***0.006***	***0.001***	*0.23*	*0.43*
Residence	Urban	163(60.6)	71(43.6)	79(48.5)	52(32)	88(54)
	Rural	106(39.4)	36(34)	41(38.7)	27(25.5)	56(52.8)
	***p-value***		*0.12*	*0.11*	*0.26*	*0.85*
BMI(Kg/m^2^)	Underweight	18(6.7)	4(22.2	6(33.3)	4(22.2)	6(33.3)
	Normal	140(52)	46(32.8)	50(35.7)	33(23.6)	72(51.4)
	Overweight	68(25.3)	27(39.7)	36(52.9)	19(27.9)	37(54.4)
	Obese	43(16)	30(69.7)	28(65.1)	23(53.5)	29(64.4)
	***P-value***		** < ** ***0.0001***	***0.002***	***0.002***	*0.087*
WC(cm)	Normal	186(69.1)	59(31.7)	66(35.5)	32(17.2)	94(50.5)
	Abnormal	83(30.9)	48(57.8)	54(65)	47(56.6)	50(60.2)
	***P-value***		** < ** ***0.0001***	** < ** ***0.0001***	** < ** ***0.0001***	*0.14*
SBP	< 130 mmHg	144(53.5)	55(38.2)	60(41.7)	42(29.2)	82(56.9)
	≥ 130 mmHg	125(46.5)	52(41.6)	60(41.7)	37(29.6)	62(49.6)
	***P-value***		*0.57*	*0.29*	*0.94*	*0.23*
DBP	< 85 mmHg	142(52.8)	54(38)	57(40.1)	41(28.8)	81(57)
	≥ 85 mmHg	127(47.2)	53(41.7)	63(49.6)	38(29.9)	63(49.6)
	***P-value***		*0.53*	*0.12*	*0.85*	*0.22*
History of smoking	Yes	46(17.1)	21(45.6) 17(36.9)		12(26.1)	17(36.9)
	No	223(82.9)	86(38.6) 103(46.2)		67(30)	127(56.9)
	***P-value***		*0.37 0.25*		*0.59*	***0.01***
Current smoking	Yes	18(6.7)	9(50)	10(55.5)	3(16.7)	12(66.7)
	No	251(93.3)	98(39)	110(43.8)	77(30.7)	132(52.6)
	***P-value***		*0.36*	*0.33*	*0.23*	*0.25*
History of alcoholism	Yes	59(21.9)	25(42.4)	33(55.9)	16(27.1)	36(61)
	No	210(78.1)	82(39)	87(41.4)	63(30)	108(51.4)
	***P-value***		*0.65*	*0.05*	*0.67*	*0.19*
Current alcoholism	Yes	28(10.4)	10(35.7)	16(57.1)	10(35.7)	17(60.7)
	No	241(89.6)	97(40.2)	104(43.1)	69(28.6)	127(52.7)
	***P-value***		*0.64*	*0.16*	*0.44*	*0.42*
Physical activity	Vigorous	26(9.6)	5(19.2)	8(30.7)	6(23.1)	9(34.6)
	Moderate	53(19.7)	17(32.1)	12(22.6)	10(18.8)	26(49)
	Low-level	102(37.9)	34(33.3)	49(48)	30(29.4)	57(55.9)
	sedentary	88(32.8)	51(57.9)	51(57.9)	33(37.5)	52(59.1)
	***P-value***		** < ** ***0.0001***	** < ** ***0.0001***	0.11	0.14
History of hypertension	Yes	148(55)	63(42.6)	68(45.9)	44(29.7)	73(49.3)
	No	121(45)	44(36.3)	52(42.9)	35(28.9)	71(58.7)
	***P-value***		*0.3*	*0.63*	*0.88*	*0.12*
History of DM	Yes	77(28.6)	30(38.9)	45(58.4)	25(32.4)	50(64.9)
	No	192(71.4)	77(40.1)	75(39)	54(28.1)	94(48.9)
	***P-value***		*0.86*	***0.004***	*0.48*	***0.018***
Family history of CVDS	Yes	105(39)	53(50.5)	52(49.5)	26(24.7)	58(55.2)
	No	164(61)	54(32.9)	68(41.4)	73(44.5)	86(52.4)
	***P-value***		**0.01**	0.34	0.12	0.96
Vegetable/fruit consumption	Low	162(60.2)	62(38.3)	70(43.2)	53(32.7)	85(52.5)
	sufficient	107(39.8)	45(42)	50(46.7)	26(24.3)	59(55.1)
	***P-value***		*0.53*	*0.57*	*0.14*	*0.67*
Hyperuricemia	Yes	116(43.1)	57(49.1)	63(53.3)	44(37.9)	72(62)
	No	153(56.9)	50(32.7)	57(37.2)	35(22.9)	72(47)
	***P-value***		0.15	***0.008***	***0.006***	***0.007***

*Abbreviations*: values are in numbers(%), *BMI* Body mass index, *CVDs* Cardiovascular diseases, *DBP* Diastolic blood pressure, *FHH* Familial history of hypertension, *FHHD* Familial history of heart disease, *DM* Diabetes mellitus, *SBP* Systolic blood pressure, *WC* Waist circumference, *TC* Total cholesterol, *HDL-c* High-density lipoprotein cholesterol, *LDL-c* Low-density lipoprotein cholesterol, *TG* Triglyceride

**Fig. 1:**
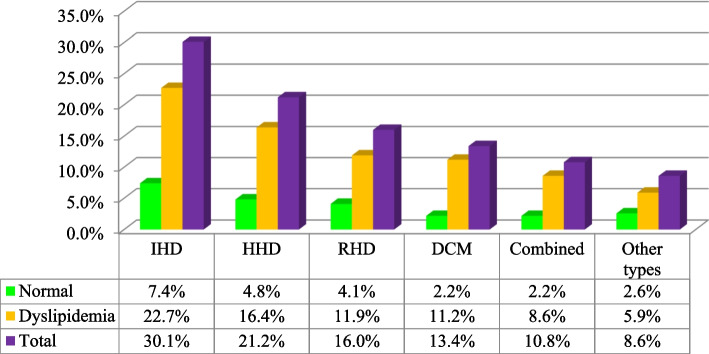
Pattern dyslipidemia among the study participants with cardiac disease type. IHD-Ischemic heart disease, HHD-Hypertensive heart disease, RHD- Rheumatic heart disease, DCM-Dilated cardiomyopathies, other types: (cor-pulmonale and degenerative heart disease (DVHD))

Dyslipidemia occurs more frequently in study participants who had ischemic heart disease, accounting for 61 (22.68%), followed by hypertensive heart disease 44 (16.36%) (Fig. 1).

### Factors associated with dyslipidemia among study participants

In bivariate analysis: being age > 54 years, the crude odds ratio (cOR) and (95%CI) was 1.9(1.2–3.2) for TC ≥ 200 mg/dl and 2.3(1.4–3.87) for TGs ≥ 150 mg/dl. Past history of smoking, the cOR and (95%CI) was 2.2(1.1–4.3) for HDL-c < 40 mg/dl; while sedentary lifestyle, the cOR and (95%CI) was 5.7(1.9–16.7) for TC ≥ 200 mg/dl, 3.1(1.2–7.8) for TGs ≥ 150 mg/dl and 2.7(1.09–6.8) for HDL-c < 40 mg/dl. In addition, a history of DM was significantly associated with TG and HDL-c dyslipidemia, while a family history of hypertension disease was significantly associated with TC and LDL-c dyslipidemia.

However, the multivariate analysis was adjusted for possible confounding factors. Therefore, age > 54 years, the adjusted odds ratio (aOR) and (95% CI) was 2.6 (1.4–4.8) for TC dyslipidemia and 2.4 (1.2–4.7) for TGs dyslipidemia. Hyperuricemia was significantly associated with LDL-c and HDL-c dyslipidemia, the aOR) and (95% CI) were 2.4(1.2–4.7) and 1.8(1.0–3.2), respectively. In addition, sedentary lifestyle and BMI ≥ 30 kg/m^2^ were significantly associated with TC dyslipidemia, the aOR) and (95% CI) were 4.5(1.4–14.6) and 6.7(1.4–32.5), respectively (Table 3).

**Table 3:** Factors associated with dyslipidemia among cardiac patients

Explanatory variable	At 95%CI	Outcome variables			
		TC ≥ 200 mg/dl	TGs ≥ 150 mg/dl	LDL-c ≥ 130 mg/dl	HDL-c < 40 mg/dl
Sex = female	cOR aOR *P*-value	1.14(0.69–1.88)	0.95(0.58–1.55)	1.5(0.9–2.6)	1.13(0.7–1.84)
		NA 0.59	NA 0.85	1.4(0.7–2.9) 0.11, 0.31*	NA 0.6
Age: > 54 years	cOR aOR *P*-value	1.9(1.2–3.2)	2.3(1.4–3.87)	1.38(0.8–2.3)	1.2(0.75–1.97)
		2.6(1.4–4.8) 0.007, 0.02*	2.4(1.2–4.7) 0.001, 0.01*	0.5(0.23–1.17) 0.23, 0.11*	NA 0.43
Residence: Urban	cOR aOR *p*-value	1.5(0.9–2.5)	1.4(0.9–2.45)	1.4(0.79–2.37)	1(0.64–1.7)
		1.2(0.6–2.3) 0.117, 0.5*	1.3(0.7–2.4) 0.11, 0.36*	NA 0.26	NA 0.85
Past alcoholism: Yes	cOR aOR *P*-value	0.87(0.48–1.56)	0.56(0.3–1.0)	1.15(0.6–2.2)	0.67(0.3–1.2)
		NA 0.64	1.9(0.9–3.86) 0.049, 0.058*	NA 0.67	1.96(0.98–3.9) 0.19, 0.057*
Current alcoholism: Yes	cOR aOR *P*-value	1.2(0.53–2.74) NA 0.64	0.57(0.25–1.2) 2.4(0.9–5.95) 0.06, 0.16*	0.7(0.3–1.6) NA 0.43	0.72(0.32–1.6) NA 0.42
Past smoking: Yes	cOR aOR *P*-value	0.75(0.39–1.42)	1.46(0.76–2.8)	1.2(0.59–2.5)	2.2(1.1–4.3)
		NA 0.37	NA 0.253	NA 0.59	0.35(0.16–0.78) 0.015, 0.001*
Currently Smoke: Yes	cOR aOR *P*-value	0.64(0.24–1.67) NA 0.36	0.6(0.24–1.63) NA 0.33	2(0.6–7.7) 0.4(0.09–1.77) 0.23, 0.24	0.55(0.2–1.52) NA 0.253
Low fruit/vegetable intake	cOR aOR *P*-value	1.17(0.7–1.93) NA 0.53	1.15(0.7–1.9) NA 0.57	1.5(0.87–2.6) 2(1.0–4.1) 0.14, 0.043*	1.1(0.68–1.8) NA 0.67
Lifestyle Sedentary:	cOR aOR *P*-value	5.7(1.9–16.7)	3.1(1.2–7.8)	2(0.7–5.5)	2.7(1.09–6.8)
		4.5(1.4–14.6) 0.001, 0.011*	1.8(0.6–5.3) 0.018, 0.26*	2.1(0.7-7.14) 0.17, 0.22*	2.8(0.96–8.2) 0.03, 0.058*
Low-level exercise:	cOR aOR *P*-value	2(0.7–6.1) 1.6(0.5–5)	2(0.8–5.2) 1.7(0.6–4.7)	1.4(0.5–3.8) NA	2.4(0.97–5.8) 2.9(1.0–8.3)
		0.17, 0.4*	0.11, 0.3*	0.52	0.057, 0.039*
History of hypertension: yes	cOR aOR *P*-value	0.77(0.47–1.26) NA 0.3	0.89(0.55–1.44) NA 0.63	0.96(0.57–1.63) NA 0.88	1.4(1.0–1.9) 0.59(0.29–1.2) 0.13, 0.59
History of DM: yes	cOR aOR *P*-value	1(0.6–1.8) NA 0.86	0.45(0.26–0.8) 1.9(1.0–3.6) 0.004, 0.048*	0.8(0.46–1.44) NA 0.48	0.5(0.3–0.89) 2(1.0–3.9) 0.018, 0.038*
FHHD: Yes	cOR aOR *P*-value	0.5(0.3–0.86)	0.8(0.48–1.29)	0.6(0.37–1.12)	0.99(0.6–1.6)
		1.9(1.1–3.5) 0.011, 0.02*	NA 0.34	0.48(0.24–0.9) 0.12, 0.03*	NA 0.96
Cardiac duration	cOR aOR *P*-value	0.9(0.8–1.05) 0.8(0.5–1.2) 0.25, 0.3*	0.94(0.84–1.1) NA 0.32	1(0.9–1.2) 1(0.8–1.22) 0.24, 0.6*	1(0.89–1.1) NA 0.98
Treatment duration	cOR aOR *P*-value	0.9(0.8–1.06) 1.1(0.7–1.76) 0.24, 0.6*	0.9(0.78–1.0) 0.8(0.67–0.94) 0.1, 0.008*	1.06(0.9–1.23) NA 0.39	1(0.87–1.14) NA 0.98
Hyperuricemia: Yes	cOR aOR *P*-value	1.9(1.2–3.27) 1.5(0.88–2.8) 0.007, 0.12	2(1.2–3.27) 1.4(0.8–2.5) 0.006, 0.21*	2(1.2–3.5) 2.4(1.2–4.7) 0.008, 0.006*	1.8(1.1–3.0) 1.8(1.0–3.2) 0.015, 0.04*
SBP: ≥ 130 mmHg	cOR aOR *P*-value	1.15(0.7–1.88)	1.3(0.79–2.1)	1(0.6–1.73)	0.74(0.46–1.19)
		NA 0.57	NA 0.29	NA 0.94	0.87(0.4–1.8) 0.23, 0.7*
DBP: ≥ 85 mmHg	cOR aOR *P*-value	1.17(0.7–1.9)	1.4(0.9–2.38)	1.05(0.62–1.78)	0.7(0.46–1.2)
		NA 0.54	1.4(0.79–2.6) 0.12, 0.23*	NA 0.85	0.78(0.39–1.58) 0.22, 0.5*
BMI:25–29.9 kg/m^2^	cOR aOR *P*-value	2.3(0.6–7.7)	2.2(0.7–6.7)	0.27(0.13–0.55)	2.3(0.8–7.1)
		1.6(0.4–6.4) 0.17, 0.4*	1.26(0.36–4.4) 0.001, 0.7*	0.98(0.21–4.64) < 0.0001, 0.98*	1.9(0.5–7) 0.12, 0.3*
BMI: ≥ 30 kg/m^2^	cOR aOR *P*-value	8(2.2–29.3)	3.7(1.1–11.9)	4(1.1–14.4)	4(1.2–13.3)
		6.7(1.4–32.5) 0.001, 0.017*	1.2(0.27–5.16) 0.2, 0.8	2.2(0.4–12.6) 0.03, 0.35*	3.3(0.7–15.4) 0.017, 0.1*
WC: ≥ 88 cm for females & ≥ 102 cm for males	cOR aOR *P*-value	2.9(1.7–5.0)	3.38(1.9–5.8)	2.7(1.58–4.8)	1.4(0.8–2.5)
		1.26(0.56–2.8) < 0.0001, 0.5*	2.6(1.16–5.8) < 0.0001, 0.02*	1.7(0.68–4.68) < 0.0001, 0.23*	0.84(0.36–1.9) 0.14, 0.6*

*Reference category:* male, age ≤ 54 years, rural, no alcoholism, no smoking, vigorous exercise, no familial history of hypertension, no history of diabetes, no familial history of heart disease, no hyperuricemia, sufficient vegetable/ fruit consumption, SBP < 130 mmHg, DBP < 85 mmHg, BMI < 25 kg/m^2^, normal WC(≤ 88 cm in females and ≤ 102 cm in males)

*Abbreviations*: *BMI* Body mass index, *DBP* Diastolic blood pressure, *FHH* Familial history of hypertension, *FHHD* Familial history of heart disease, *DM* Diabetic Mellitus, *SBP* Systolic blood pressure, *WC* Waist circumference, *TC* Total cholesterol, *HDL-c* High-density lipoprotein cholesterol, *LDL-c* Low-density lipoprotein cholesterol, *TG* Triglyceride, *cOR* Crude odd ratio, *aOR* Adjusted odds ratio, *NA* Not applicable; ^*^, the *p*-value for adjusted odds ratio; mmHg, millimeter of mercury, *kg* kilogram; m, meter

### Correlations of lipid profile with the explanatory variables among the study participants

TC, TG and LDL-c were positively correlated with WC and BMI, while TGs and LDL-c were positively correlated with age. In addition, TC and TGs were positively correlated with uric acid. However, TGs was negatively and significantly associated with cardiac disease duration (Table 4).

**Table 4:** Correlation of dyslipidemia with independent variables of cardiac patients

Variables	Mean ± SD	Significance	TC	TG	LDL-c	HDL-c
Age	51.13 ± 15.8	Correlation coefficient(r) *p*-value	0.116 0.057	0.19 0.002	0.149 0.015	-0.02 0.968
Cardiac duration	3.9 ± 2.1	Correlation coefficient (r)	-0.056	-0.089	0.108	0.014
		*p*-value	0.364	0.144	0.076	0.818
Treatment duration	3.23 ± 1.8	Correlation coefficient (r)	-0.057	-0.126	0.066	-0.16
		*p*-value	0.351	0.038	0.28	0.795
SBP	132.8 ± 14.9	Correlation coefficient *p*-value	0.078 0.204	0.09 0.139	0.47 0.439	-0.16 0.792
DBP	85.5 ± 7.4	Correlation coefficient (r)	0.063	0.07	0.00	-0.005
		*p*-value	0.305	0.252	1.00	0.938
waist circumference	91.4 ± 10.4	Correlation coefficient (r)	0.296	0.226	0.219	-0.088
		*p*-value	< 0.001	< 0.001	< 0.001	0.15
BMI	24.4 ± 4	Correlation coefficient (r)	0.334	0.263	0.280	-0.033
		*p*-value	< 0.001	< 0.001	< 0.001	0.589
Uric acid	6.2 ± 1.92	Correlation coefficient *p*-value	0.193 0.001	0.188 0.002	0.105 0.086	-0.007 0.911

*DBP* Diastolic blood pressure, *SBP* Systolic blood pressure, *BMI* Body mass index, *SD* Standard deviation

## Discussion

Dyslipidemia is the most important independent predictor of cardiovascular disease, leading to high morbidity and mortality in cardiac patients. Therefore, this cross-sectional study was conducted in a resource-constrained East African setting to determine the burden of dyslipidemia and its risk factors in cardiac patients.

In this study, the overall prevalence of dyslipidemia was 76.6% (95% CI: 72,181,0) using the NCEP ATP III criteria and the rate was almost comparable with the study reported from Iran, which indicated that 78.9% of cardiac patients suffered from dyslipidemia [24]. However, it is not consistent with the studies conducted in Saudi Arabia [25], Cameroon [1] and Gondar, Ethiopia [17], where the prevalence was 47.9%, 56.4% and 34%, respectively. Patient lifestyle, sample size, classification criteria for dyslipidemia, and genetic variability in the population may account for the differences described.

In this study, dyslipidemia was higher in males (57.3%) than in females (42.7%). Conversely, the study reported from Iran [24], indicated that the rate of dyslipidemia was higher in females as compared to males. This discrepancy could be due to the fact that almost 57% of participants in the current study were males and also it might be related to the experiences of smoking and alcoholism.

In this study, the most frequently observed dyslipidemia was low HDL-c (< 40 mg/dl), with a rate of 53.5%. This is almost comparable to the study conducted in Qatar, which was 56.8% [26]. However, the result was different from the studies conducted in Cameroon [1] and Egypt [27], where the rates were 44.3% and 66%, respectively. The use of lipid-lowering drugs in some study participants [1], while study design, the degree of urbanization, and lifestyle [27] could be some reasons for the inconsistency of HDL-c dyslipidemia rates between the studies.

In this study, the prevalence of TGs dyslipidemia was 44.6%. It was inconsistent with the rate reported from different studies: like 63% among CHD patients in India [28], 63.3% in Egypt [27], 39.7% in Spain [29], 31% in Malaysia [30] and 18.7% in Cameroon [1]. However, the use of lipid-lowering drugs in some studies, the extent of lipid profile disturbances caused by different cardiac treatment agents, duration of cardiac treatment, dietary habits and location could show a variation in the prevalence rates between studies.

In addition, the prevalence of high TC in our study was 39.8%, which was higher than the study conducted in India, 23.3% [28] and Cameroon, 3.4% [1]. However, the finding was lower than the study done in Malaysia, 51% [30]and Egypt, 60.6% [27]. The variation might be due to anti-cholesterol drug utilization in Cameroon study participants, genetic disparity, lifestyle, and the study design.

The prevalence of high LDL-c was 29.4% which was higher than the studies reported in Malaysia, 9% [30], and Cameroon, 3.8% [1], but lower than the study done in Egypt, 58% [27]. The difference may be attributed to the method used to classify dyslipidemia, lifestyle, physical inactivity, and genetic disparity.

In addition, in this study, the prevalence of TC dyslipidemia was 39.8% and this is not in line with the studies conducted in India [28], Malaysia [30], and Egypt [27], where the prevalence was 23.3% 51% and 60.6%, respectively. A sedentary lifestyle and genetic variability in the population could be a reason for the differences described. Further, in this study, TC and TG dyslipidemia was higher in males than females. This finding is consistent with the study conducted in Egypt, where TC and TG dyslipidemia was significantly higher in males [27]. In support of the current finding, the study conducted in India [28], also suggested that male participants were more likely to suffer from dyslipidemia than females.

Regarding risk factors, the present study showed that TC and TG dyslipidemia is significantly and positively associated with age > 54 years. This could be due to an age-related increase in postprandial dyslipidemia, insulin resistance, and age-related decline in ApoB/E receptor and sex hormones (estrogen and androgen) [31]. In this study, a sedentary lifestyle was significantly associated with TC dyslipidemia. However, studies have shown that individuals who engage in performing moderate to vigorous physical activity protect against cardiovascular disease by improving endothelial function, reducing insulin resistance, reducing obesity, and increasing HDL-c levels [32, 33].

The present study showed that sex, ethnicity, residence, alcohol consumption and hypertension have no significant effect on lipid profile (*p* > 0.05), which was comparable with the study reported from Sudan [34]. However, a FHHD was associated with TC and LDL-c dyslipidemia, while previous smoking was significantly associated with HDL-c dyslipidemia. This is not in line with the study conducted in Sudan. The variation may be due to differences in genetic predisposition beteen the population, practicing unhealthy behavior, and study design between the studies.

In this study, obesity was associated with a higher risk of having hypercholesterolemia and the finding was in line with the study conducted in the Iranian adult population [35]. This is because obesity increases the likelihood of having elevated cholesterol level, which is triggered by insulin resistance, adipokines, and free fatty acids [36, 37]. In this study, low fruit/vegetable consumption was associated with LDL-c dyslipidemia in cardiac patients. In support, a study found that fruit and vegetable consumption was inversely related to LDL-c in both men and women [38]. The possible explanation for this may be, diets high in fruits and vegetables can protect against dyslipidemia because vitamins, minerals, and other multiple nutritional factors in fruits and vegetables may decrease inflammation and oxidative stress, insulin sensitivity, and blood pressure [39].

We found that DM is significantly associated with dyslipidemia among cardiac patients and this is a common phenomenon in patients with type 2 diabetes because insulin resistance or insulin deficiency impairs important enzymes and pathways in lipid metabolism [40].

Furthermore, studies revealed that the association of hyperuricemia with dyslipidemia [41, 42]. Similarly, we found a significant association of hyperuricemia with HDL-c and LDL-c dyslipidemia.

### Limitations of the study

Firstly, we conducted a cross-sectional study, which cannot provide adequate evidence of causation regarding dyslipidemia and its risk factors. Secondly, we have not compared the findings with the control group because this study was conducted in the hospital, most people visit the hospital for medical purposes and it is difficult to get a sufficient number of apparently healthy subjects as a control group. Regardless of the depicted limitations, this study provides helpful information in the scarce data situation of Ethiopia.

## Conclusion

The results of this study showed that there was a high prevalence of dyslipidemia in cardiac patients based on the NCEP-ATP-III criteria. The most commonly observed dyslipidemia was low HDL-c, followed by TG, TC, and LDL-c dyslipidemia. In addition, older age, presence of diabetes, smoking habits, vegetable/fruit consumption status, body weight, abdominal obesity, and a FHHD, sedentary lifestyle, duration of treatment, and hyperuricemia are significantly associated with dyslipidemia. Therefore, regular assessment of lipid profiles is crucial to minimize further complications from dyslipidemia among patients with cardiac disease. In addition, intensive counseling of the patients with regard to physical activity, behavior change and diet modification is also mandatory. Furthermore, well controlled and large scale longitudinal studies are recommended to address other unexplained factors of dyslipidemia and hidden burden of dyslipidemia among cardiac patients at regional or national level.

## Data Availability

The dataset of this article is not openly available but it can be accessed on reasonable requests from the corresponding author.

## Abbreviations

aOR: Adjusted odds ratio
BMI: Body mass index
BP: Blood pressure
CI: Confidence interval
cOR: Crude odds ratio
CVD: Cardiovascular diseases
HDL-c: High density lipoprotein-cholesterol
NCEP-ATP: National Cholesterol Education Program-Adult Treatment Panel
SPSS: Statistical package for social sciences
TG: Triglycerides
WHO: World Health Organization

## References

[1] Ama Moor VJ, NdongoAmougou S, Ombotto S, Ntone F, Wouamba DE, Ngo Nonga B (2017). Dyslipidemia in patients with a cardiovascular risk and disease at the university teaching hospital of yaoundé. Cameroon. Int J Vasc Med.

[2] Labarthe DR (2011). Epidemiology and Prevention of Cardiovascular Diseases : A Global ChaLLEnge second edition. second. Michael Brown.

[3] Deaton C, Froelicher ES, Wu LH, Ho C, Shishani K, Jaarsma T (2011). The global burden of cardiovascular disease. Eur J Cardiovasc Nurs.

[4] World Health Organization (WHO) (2021). Cardiovascular diseases.

[5] Abdosh T, Weldegebreal F, Teklemariam Z, Mitiku H (2019). Cardiovascular diseases risk factors among adult diabetic patients in eastern Ethiopia. JRSM Cardiovasc Dis.

[6] Rehan F, Qadeer A, Bashir I, Jamshaid M (2016). Risk factors of cardiovascular disease in developing countries. Int Curr Pharm J.

[7] Nelson RH (2013). Hyperlipidemia as a risk factor for cardiovascular disease. Prim Care.

[8] Oguejiofor OC, Onwukwe CH, Odenigbo CU (2012). Dyslipidemia in Nigeria : Prevalence and pattern. Ann African Med.

[9] Reiner PŽ, Ph D (2016). Classification of cardiovascular diseases caused by atherosclerosis. Int Fed Clin Chem Lab Med.

[10] Mahalle N, Garg MK, Naik SS, Kulkarni MV (2014). pattern of dyslipidemia and its correlation with cardiovascular risk factors in patients with proven coronary artery disease. J Endocrinol Metab.

[11] Nepal G, Tuladhar ET, Acharya K, Bhattarai A, Sharma VK, Raut M, et al. Dyslipidemia and associated cardiovascular risk factors among young Nepalese university students. Cureus. 2018;10(1):e2089. 10.7759/cureus.2089 PMC5860887 29564194

[12] Perry Wengrofsky JL and ANM.Dyslipidemia and Its Role in the Pathogenesis of Atherosclerotic Cardiovascular Disease. Intech open. 2019;11:1–19.

[13] Yao YS, Di LT, Zeng ZH (2020). Mechanisms underlying direct actions of hyperlipidemia on myocardium: an updated review. Lipids Health Dis.

[14] Fakhrzadeh H, Tabatabaei-Malazy O, Kelishadi R (2012). Dyslipidemia and cardiovascular disease. Dyslipidemia: from prevention to treatment.

[15] Yadeta D, Walelgne W, Fourie JM, Scholtz W, Nel G, Scarlatescu O (2021). PASCAR and WHF Cardiovascular Diseases Scorecard project. Cardiovasc J Afr.

[16] Tefera YG, Abegaz TM, Abebe TB, Mekuria AB (2017). The changing trend of cardiovascular disease and its clinical characteristics in Ethiopia. Vasc Health Risk Manag.

[17] Atkilt G, Shaweno T, Disease CH, Disease HH (2019). Determinants of hypertensive heart disease among adult hypertensive patients in the University of Gondar Referral Hospital, Gondar, Ethiopia: A case-control study. Cardio Thorac Med.

[18] Shashu BA, Ayele MA (2014). The pattern of coronary artery diseases as diagnosed by coronary angiography and the outcome of Percutaneous Coronary Intervention ( PCI ) in Ethiopia. Ethiop J Heal Dev.

[19] (WHO): WHO. Chronic diseases and health promotion: Stepwise approach to surveillance (STEPS). 2010. http://www.who.int/chp/steps/manual/en.

[20] Asaye S, Bekele S, Tolessa D, Cheneke W (2018). Metabolic syndrome and associated factors among psychiatric patients. Diabetes Metab Syndr Clin Res Rev.

[21] National Cholesterol Education Program (NCEP) Expert Panel on Detection, Evaluation, and Treatment of High Blood Cholesterol in Adults (Adult Treatment Panel III). Third report of the National Cholesterol Education Program (NCEP) expert panel on detection, evaluation, and treatment of high blood cholesterol in adults (Adult Treatment Panel III) final report. Circulation. 2002;106(25):3143–421. 12485966

[22] Cho J, Kim C, Kang DR, Park JB (2016). Hyperuricemia and uncontrolled hypertension in treated hypertensive patients: K-MetS Study. Med (United States).

[23] Kebede B, Ayele G, Haftu D, Gebremichael G (2020). The prevalence and associated factors of hypertension among adults in Southern Ethiopia. Hindawi Int J Chronic Dis.

[24] Hedayatnia M, Asadi Z, Zare-Feyzabadi R, Yaghooti-Khorasani M, Ghazizadeh H, Ghaffarian-Zirak R (2020). Dyslipidemia and cardiovascular disease risk among the MASHAD study population. Lipids Health Dis.

[25] Alrahimi J, Alattas R, Almansouri H, Alharazi GB, Mufti HN (2020). Assessment of different risk factors among adult cardiac patients at a single cardiac center in Saudi Arabia. Cureus.

[26] Zainel AA, Al Nuaimi AS, Syed MA, A/Qotba HA. Risk factors associated with cardiovascular diseases among adults attending the primary health care centers in Qatar, a cross-sectional study. J Community Med Public Health. 2020;4:171.

[27] Abdelaziz A, Fawzy M (2014). Prevalence and pattern of dyslipidemia in acute coronary syndrome patients admitted to medical intensive care unit in Zagazig University Hospital. Egypt Zagazig Univ Med J.

[28] Mahalle N, Garg M, Naik S, Kulkarni M (2014). Study of pattern of dyslipidemia and its correlation with cardiovascular risk factors in patients with proven coronary artery disease. Indian J Endocrinol Metab.

[29] Lahoz C, Mostaza JM, Tranche S, Martin-jadraque R, Sanchez-zamorano MA, Taboada M (2012). Atherogenic dyslipidemia in patients with established coronary artery disease. Nutr Metab Cardiovasc Dis.

[30] Haque ATME, Yusoff FBM, Bin Ariffin MHS, Bin Ab Hamid MF, Hashim SRB, Haque M. Lipid profile of the Coronary heart disease (CHD) patients admitted in a hospital in Malaysia. J Appl Pharm Sci. 2016;6(5):137–42.

[31] Liu H, Li J (2015). Aging and dyslipidemia : A review of potential mechanisms. Elsevier Sci direct.

[32] World Health Organization (WHO) (2011). Global Atlas on cardiovascular disease prevention and control, Geveva.

[33] Nutrition B, Force FT. Cardiovascular Disease Diet, Nutrition and Emerging Risk Factors. second edi. Frayn, K. N. (Keith N.) | Stanner, Sara, and Coe S, editor. Blackwell; 2019. p: 1–311.

[34] Musa HH, Tyrab EMA, Hamid MMA, Elbashir EAR, Yahia LM, Salih NM (2013). Characterization of lipid profile in coronary heart disease patients in Sudan. Indian Heart J.

[35] Veghari G, Sedaghat M, Moharloei P (2013). Obesity and risk of hypercholesterolemia in Iranian northern adults. ARYA Atheroscler.

[36] Klop B, Elte JWF, Cabezas MC (2013). Dyslipidemia in Obesity: Mechanisms and Potential Targets. Nutrients.

[37] Manawat R, Sharma VK (2020). Association of anthropometric variables with dyslipidemia in obesity. Physiol Pharm Pharmacol.

[38] Heart F, Djoussé L, Arnett DK, Coon H, Province MA, Moore LL (2004). Fruit and vegetable consumption and LDL cholesterol : the National. Am J Clin Nutr.

[39] Kret J, Gebeyehu S, Diggs-outlaw R, Disease C, Programs D (2014). The Burden of Cardiovascular Disease in the District of Columbia.

[40] Mithal A, Majhi D, Shunmugavelu M, Talwarkar PG, Vasnawala H, Raza AS (2014). Original Article Prevalence of dyslipidemia in adult Indian diabetic patients : a cross sectional study ( SOLID ). Indian J Endocrinol Metab.

[41] Hong JW, Noh JH, Kim DJ (2020). Association between serum uric acid and spirometric pulmonary function in Korean adults: The 2016 Korea National Health and Nutrition Examination Survey. PLoS One.

[42] Chen S, Yang H, Chen Y, Wang J, Xu L, Miao M (2020). Association between serum uric acid levels and dyslipidemia in Chinese adults. A cross-sectional study Furth meta-analysis. J Med.

